# Analysis of Decision-Making Process Using Methods of Quantitative Electroencephalography and Machine Learning Tools

**DOI:** 10.3389/fninf.2019.00073

**Published:** 2019-11-27

**Authors:** Grzegorz M. Wojcik, Jolanta Masiak, Andrzej Kawiak, Lukasz Kwasniewicz, Piotr Schneider, Filip Postepski, Anna Gajos-Balinska

**Affiliations:** ^1^Chair of Neuroinformatics and Biomedical Engineering, Faculty of Mathematics, Physics and Computer Science, Institute of Computer Science, Maria Curie-Sklodowska University, Lublin, Poland; ^2^Neurophysiological Independent Unit of the Department of Psychiatry, Medical University of Lublin, Lublin, Poland

**Keywords:** electroencephalography, sLORETA, psychiatric disorders, frequency band analysis, machine learning, Iowa Gambling Task, decision-making

## Abstract

The electroencephalographic activity of particular brain areas during the decision making process is still little known. This paper presents results of experiments on the group of 30 patients with a wide range of psychiatric disorders and 41 members of the control group. All subjects were performing the Iowa Gambling Task that is often used for decision process investigations. The electroencephalographical activity of participants was recorded using the dense array amplifier. The most frequently active Brodmann Areas were estimated by means of the photogrammetry techniques and source localization algorithms. The analysis was conducted in the full frequency as well as in alpha, beta, gamma, delta, and theta bands. Next the mean electric charge flowing through each of the most frequently active areas and for each frequency band was calculated. The comparison of the results obtained for the subjects and the control groups is presented. The difference in activity of the selected Brodmann Areas can be observed in all variants of the task. The hyperactivity of amygdala is found in both the patients and the control group. It is noted that the somatosensory association cortex, dorsolateral prefrontal cortex, and primary visual cortex play an important role in the decision-making process as well. Some of our results confirm the previous findings in the fMRI experiments. In addition, the results of the electroencephalographic analysis in the broadband as well as in specific frequency bands were used as inputs to several machine learning classifiers built in Azure Machine Learning environment. Comparison of classifiers' efficiency is presented to some extent and finding the most effective classifier may be important for planning research strategy toward finding decision-making biomarkers in cortical activity for both healthy people and those suffering from psychiatric disorders.

## Introduction

Decision-making is an essential skill in everyday life but currently there is little systematic knowledge about how decision-making is affected in people with a diagnosis of psychiatric disorders. Decision-making is a process in which many cognitive functions are engaged. Probably that is why the IGT was often chosen as a task for investigating the behavior of the people with psychiatric disorders, however, there is relatively not much known about the cortical activity of individuals while making decisions in both healthy people and those with psychiatric disorders diagnosis. Some research has been done on the patients with major depressive disorder (Cella et al., 2010; Brevers et al., 2013). Similarly, the cohort of subjects with borderline personality disorder was investigated using IGT (Haaland and Landrø, 2007) as well as bipolar disorder (Paulus, 2007). IGT applications for a variety of research and different disorders are presented to some extent in a review by Brevers et al. (2013) and originally in Bechara (2007). With many applications in psychiatry, we decided to choose the IGT out of many other tasks for this stage of our research.

Quantitative electroencephalography is at its Renaissance stage in last decades (Sand et al., 2013) and has developed toward some forms of research in modern psychiatry (Kamarajan and Porjesz, 2015; Martínez-Rodrigo et al., 2017).

The rapid increase in the number of publications concerning Brain-Computer Interfaces (BCI) is observed (Mikołajewska and Mikołajewski, 2012, 2013, 2014; Teruel et al., 2017; Ozga et al., 2018; Wierzgała et al., 2018) and the EEG activity can be recognized as one of possible solutions in BCI engineering (Kotyra and Wojcik, 2017a,b). In addition, any ideas for finding biomarkers of psychiatric disorders (Chapman and Bragdon, 1964; Sutton et al., 1965; Campanella, 2013; Golonka et al., 2017) are in demand as the interview is still the most often used tool in psychiatry to make the diagnosis.

The expansion of computational modeling techniques applied to neuroscience makes it possible to simulate selected parts of the brain tissues which we are familiar with (Wojcik et al., 2007; Wojcik and Kaminski, 2008; Wojcik and Garcia-Lazaro, 2010) or even investigate the influence of electrophysiological parameters of single cells on the dynamics of the whole simulated system (Wojcik and Kaminski, 2007; Wojcik, 2012). However, we are still very far from explaining complex phenomena like psychiatric disorders or syndromes e.g., burn-out (Chow et al., 2018). Higher cognitive functions are sometimes a source of inspiration in biomedical engineering (Kaminski and Wojcik, 2004; Ważny and Wojcik, 2014; Wojcik and Ważny, 2015; Kufel and Wojcik, 2018) and artificial intelligence (Ogiela et al., 2008; Szaleniec et al., 2008, 2013) mixed with cognitive science methodology provides some explanation or leads to the construction of classification tools. Nevertheless, we are still in demand for verification theory in the experiment.

There are different electroencephalographic methods that allow visualization of recorded activity on the brain model. One of them is the standardized low-resolution brain electromagnetic tomography algorithm (sLORETA) (Pascual-Marqui et al., 1994, 1999; Pascual-Marqui, 2002). This method advantages come from the high temporal resolution of modern electroencephalographs (Tohka and Ruotsalainen, 2012) and makes possible to compute the subjects brain activity distributed in time and put it on brain topography with the tomography-like quality of detail. Applications of sLORETA were reported e.g., for the attention-deficit-hyperactivity disorder (ADHD) (Mann et al., 1992) and neurodegenerative diseases (Wu et al., 2014). The sLORETA can be also applied in the frequency band analysis (Moretti et al., 2004; Saletu et al., 2010).

Using EEG based source localization techniques for the measurement of subcortical activity can be controversial. We are aware of the fact that for example in Krishnaswamy et al. (2017) authors state that subcortical structures produce smaller scalp EEG signals. This happens because they are farther from the head surface than cortical structures. To make matters worse, subcortical neurons can have a closed-field geometry that further weakens the observed distant fields and subcortical structures are surrounded by the cortical mantle. So measurements of activity in deep brain structures can potentially be explained by a surrogate distribution of currents on the cortex. That is why it can be very difficult to measure subcortical activity when cortical activity is occurring at the same time (Krishnaswamy et al., 2017). However, there are various mathematical models (Grech et al., 2008) that allow us to make some estimation of such kind of activity. Our lab is equipped with the very sophisticated and developed for 25 years GeoSource software^1^, where such models are implemented and based on the results given by it, having access to the photogrammetry station which generates the head model with high accuracy, we are able to draw some conclusions that are some extrapolated indicators for subcortical areas increased activity. The GeoSource is not the only software with subcortical areas activity algorithmic detectors. We have done some comparative analysis with BESA and its: ERP analysis and averaging^2^ and source analysis and imaging^3^ packages getting the same quality of results.

The investigations of Event-Related Potentials are often chosen by experimental psychologists as well as clinicians and biomedical engineers. One of the best-recognized ERP experiments in which decision-making is investigated was proposed by Bechara et al. (1994). It is known as the Iowa Gambling Task (IGT) and is described in detail in the Materials and Methods section of this contribution.

IGT was used in many clinical experiments (Cui et al., 2013; Mapelli et al., 2014; Tamburin et al., 2014). In Tamburin et al. (2014) the patients with chronic low back pain were investigated and the authors tried to find correlations between the ERP responses and the cognitive measures taken on them. On the other hand, in Cui et al. (2013) the students were investigated during IGT and the amplitudes of P3 potential were observed and discussed. Similar research is reported (Mapelli et al., 2014) but in this case it was focused on the people with Parkinson's disease making decisions and after that their ERP potentials were analyzed. The research mentioned above is concentrated on the analysis of the shape of statistically averaged potential and there are no source localization procedures applied to the analysis.

The aim of the research presented herein was to apply the methodology proposed in Wojcik et al. (2018a) and Wojcik et al. (2018b) to the quantitative electroencephalographic analysis of cortical activity from the patients in different frequency bands as well as in the full spectrum of the EEG signal. We used source localization techniques and having measured the average amperage in time for particular Brodmann Areas (BA) the mean electric charge flowing through them during the experiment was conducted for each patient and member of the control group. For this contribution, the brain activity of a group of patients with selected psychiatric disorders was measured using dense array EEG. These results were compared with those obtained from the participants of the control group. Both groups performed IGT.

Additionally, the results gathered for both healthy and disordered people in the broad and particular frequency EEG bands were taken as inputs to seven different machine learning classifiers in order to distinguish two types of responses in IGT, basing only on BA activity. The efficiency of these classifiers was compared and is presented to some extent.

## Materials and Methods

The Department of Neuroinformatics is equipped with the dense array amplifier recording the cortical activity with up to 500 Hz frequency through 256 channels HydroCel GSN 130 Geodesic Sensor Nets provided by EGI^4^. In addition, in the EEG Laboratory the Geodesic Photogrammetry System (GPS) was used. Eleven cameras placed in the corners of GPS take a set of subject's photos and then it is possible to make a model of the particular subject brain based on its calculated size, proportion and shape. Next the software imposes all computed activity results on this model with a very good accuracy. The amplifier operates on the Net Station 4.5.4 software, GPS is under control of the Net Local 1.00.00 and GeoSource 2.0. The eye blinks and saccades elimination as well as gaze calibration are obtained owing to the application of dedicated eye-tracker operated by SmartEye 5.9.7. The Event-Related Potentials (ERP) experiments are conducted in the PST e-Prime 2.0.8.90 environment^5^.

We investigated 30 patients, 9 females and 21 males (avg. age 28.1, s.d. 12.4). They have been diagnosed with a wide range of psychiatric disorders. The disorders are classified in ICD-10 as: 12 × F41 (Panic disorder), 5 × F32.1 (Major depressive episode), 5 × F84.5 (Asperger syndrome), 3 × F40 (Social anxiety disorders), 2 × F31 (Bipolar affective disorder), 2 × F42 (Obsessive-compulsive disorder co-occurrent with the patients with F84.5), 2 × F51.1 (Non-organic hypersomnia), and 1 × F20 (Schizophrenia). The control group of 30 healthy volunteers were also examined. The control group were only males (avg. age 22.4, s.d. 1.7). It is worth noting that about 30% more subjects were investigated from both control and patients' groups as the signal of all those for whom the recordings were too noisy or incomplete had to be eliminated. All participants were right-handed and measured by a handedness questionnaire (Chapman and Chapman, 1987).

**Figure 1:**
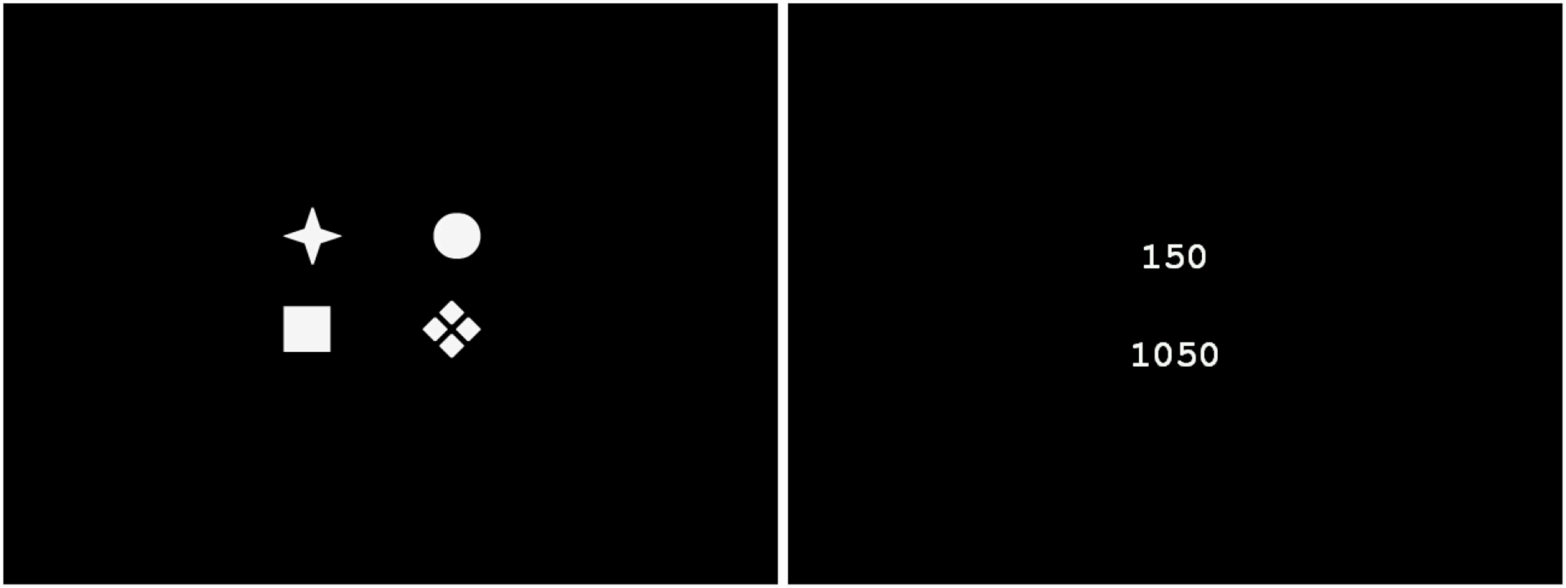
Typical screens shown to participants during the IGT experiment. Card decks—in the left and reward/punishment with account state in the right. The screens are generated by the PST e-Prime 2.0.8.90 which is synchronized with Net Station 4.5.4 recordings.

The IGT was introduced by Bechara et al. (1994) and since then it has become one of the favorite tasks given to the subjects participating in a wide range of experimental psychology experiments. Originating from the research first carried out at the University of Iowa the IGT was intended to get hold of mechanisms of decision-making process during the reward-punishment oriented card game. The aim of the task is to choose one card deck symbol out of four in each of 100 trials. The participants are told to earn as much of virtual money as possible starting with 0 dollars. In each set of four cards (or symbols) there is a couple of so-called good cards for which there is a reward and a couple of so-called bad cards for which there is a punishment. The participants do not know which card is good and which is bad but they can conclude it from the game behavior. However, at the beginning all cards seem to be good, but for two of them they make impression to be better as the reward for choosing them is remarkably higher than for choosing the others. After several choices of the better cards, the punishments for choosing the next are extremely high. On the other hand the punishment for choosing cards after the initial selection of those worse at the beginning is very low which finally gives the better financial results when compared to the other case. The typical screens shown on the computer on which our participants make decisions is shown in Figure 1.

The electroencephalographic signal was recorded. After the test the photo of each participant was taken using the GPS. Such a technique allows obtaining spatial resolutions comparable to 1.5 T MRI without the necessity of MRI brain scanning for each participant. Thus, the anatomical models of participants' brains are generated using the GeoSource software and GPS photogrammetry which allows us to estimate the activity of particular BAs with satisfactory precision. Note, that in our approach we do not use the default model of the head, which is also possible. We make use of GPS to achieve the best possible accuracy of source localization.

Such methodology allowed us to apply the source localization algorithm with a satisfactory accuracy and estimate the most active Brodmann Areas in each participant during the decision-making process. The Net Station software along with the GeoSource tool has implemented the most popular version of the sLORETA algorithm which is described in the chapter titled Brain Source Localization Using EEG Signals in Nidal and Malik (2014). It is based on standardization of the current density assumption. That means that both the variance of the noise in the signal and the biological variance in the actual signal are taken into account (Goldenholz et al., 2009; Nidal and Malik, 2014). Independent and uniform distribution of the biological signal variance across the brain cortex is taken into consideration and this results in a linear imaging localization technique having exact, zero-localization error (Goldenholz et al., 2009; Nidal and Malik, 2014). For more details see the comparison of different types of LORETA in Nidal and Malik (2014).

The literature reports a few bands that cover typical rhythmical activity of the brain (Niedermeyer and da Silva, 2005) described as follows: δ—delta band (<4 Hz), θ—theta (4–7 Hz), α—alpha (8–15 Hz), β—beta (16–31 Hz), γ—gamma (more than 31 Hz), and sometimes μ—mu (8–12 Hz) bands. Sometimes the frequency ranges that define each band are slightly different. In our lab by default the frequency bands are set as follow: δ—delta band (0.1–3 Hz), θ—theta (4–7 Hz), α—alpha (7–12 Hz), β—beta (12–30 Hz), γ—gamma (more than 32 Hz).

**Figure 2:**
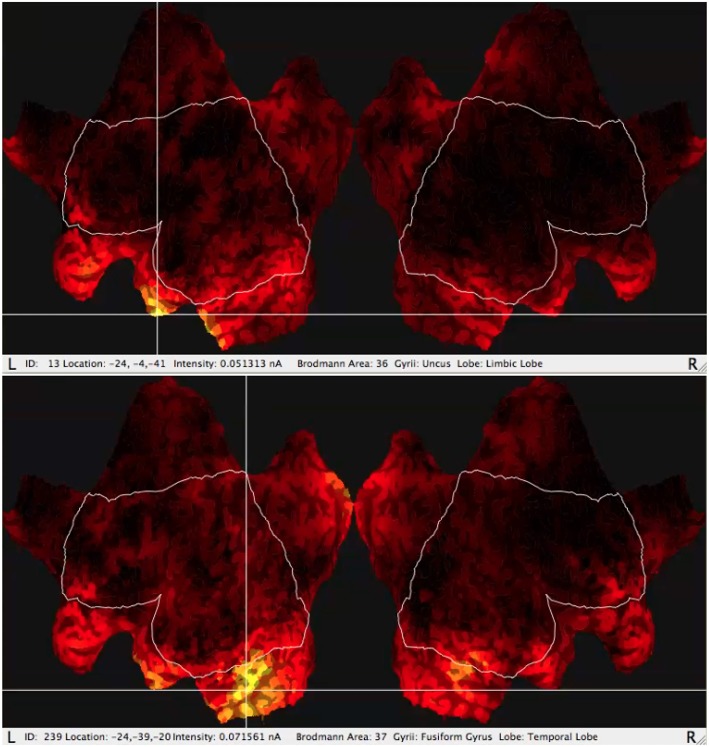
Typical results of GeoSource BA activity visualization on the brain cortex so-called Flat Map. The increase of activity in BA36 (Uncus Lobe, Limbic Lobe) for good choice and BA37 (Fusiform Gyrus, Temporal Lobe) for bad choice are indicated.

One of the most useful functions of the GeoSource software is the possibility of estimation of the amperage of the most active areas (Figure 2) varying in time using source localization. The most active BA is indicated by the GeoSource as the BA with the highest electric current flowing through it in time. The activity of a particular BA could last at its maximum value for a longer or shorter period and it could appear more than once during each epoch. The signal was divided into epochs, as usual in ERP, in this case, IGT experiments, then averaged giving amperage in the function of time. Based on the electrical current measured by the EEG amplifier, most active BAs precisely indicated by the photogrammetry station and having precisely estimated time intervals owing to the perfect EEG time resolution, one of many numerical methods for integration can be applied to calculate the mean electric charge ι with good precision (Wojcik et al., 2018a) by integrating the electrical current in time. The details of mean electric charge estimation are described in detail in Wojcik et al. (2018a).

The sLORETA can be run for the full EEG frequency band above 0.1 Hz including the γ spectrum and for the selected frequency band analysis. Besides the full band there were taken into consideration each of the following: alpha, beta, gamma, delta, and theta. For each band including the full band, the varying in time value of amperage of particular BAs was obtained from the GeoSource. Having the amperage in the function of time one can calculate the mean electric charge ι flowing through the given BA as described in Wojcik et al. (2018a). The typical visualization of the GeoSource application to the signal is shown in the flat maps in Figure 2.

The time interval in which the BA activity was calculated was set to 5 ms and there was chosen the 800 ms segmentation (each segment starting with the stimuli) for signal averaging.

The BA1, BA2, and BA3 were eliminated from our analysis as they are part of Primary Somatosensory Cortex (S1) which was hyperactive owing to the subject's fingertips contact with the response pad during the experiment.

**Figure 3:**
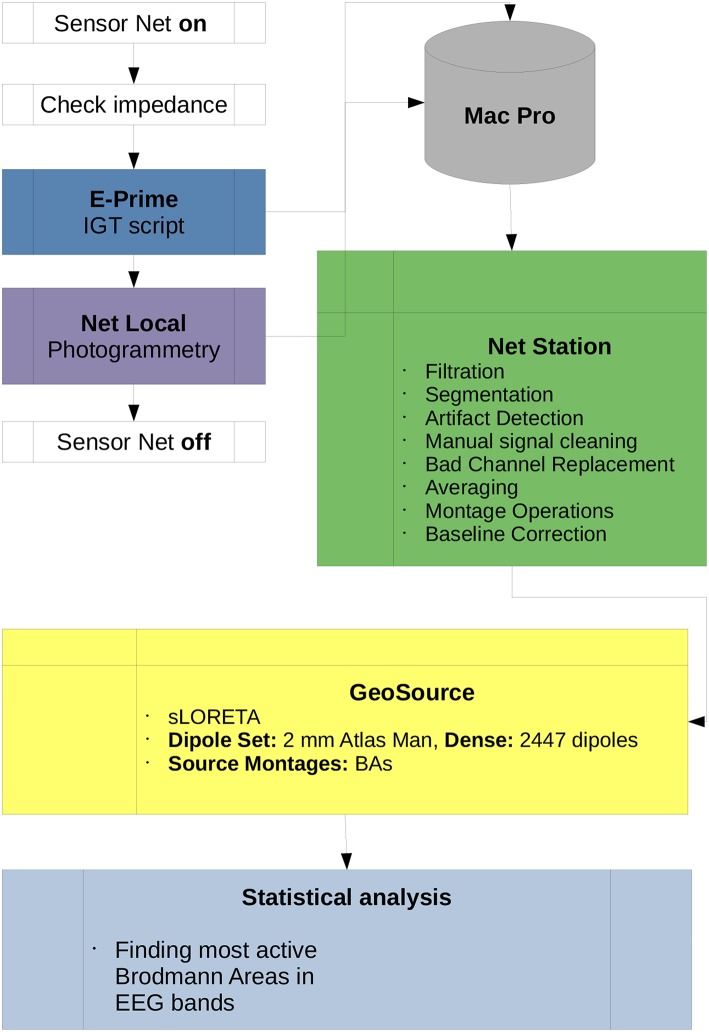
Diagram of the IGT research protocol used in this paper. All scripts used for the preprocessing data in Net Station and post-processing in GeoSource are listed. Participation of the subject in the experiment begins when the Sensor Net is put on and ends when it is taken off. All data is collected by the Mac Pro workstation which is the central computational unit of the lab. Statistical analysis, finding the most active BAs in full or each of α, β, γ, δ, and θ frequency bands as well as ι estimations can be conducted on other machines.

The scheme of the methodology and research protocol are presented in Figure 3. The full band analysis protocol in the case of P300 experiments was presented in Wojcik et al. (2018a) and the frequency band analysis protocol was described in detail in DIGITS related paper in Wojcik et al. (2018b). For this contribution the mixture of both methods proposed in Wojcik et al. (2018a,b) is applied.

The so-called Waveform Tools package from the Net Station ecosystem was used and all scripts shown in Figure 3 originate from it. The description of algorithms used in the preprocessing and post-processing stages of the research is given in Electrical Geodesics (2003) and the procedures used in the photogrammetry Net Local are described in the EGI Lab documentation (Electrical Geodesics, 2009, 2011). There were 100 trials for each participant, duration of the experiment was around 12 min. For the preprocessing we used the following and suggested by EGI engineers rules: As an average reference the average of all electrodes was taken. The artifact correction parameters were set as follow: bad channels filtering—Max-Min >200 μV; eye-blinks—Max-Min >140 μV, eye movements—Max-Min >140 μV—all performed in moving average of 80 ms. Filtration settings were set to passband gain 99%, stopband gain 1% and rollof 2 Hz, The segmentation was performed from 100 ms before stimulus to 700 after stimulus with offset 13 ms. The baseline correction was set to 100 ms from portion of segment. The rejection of trials took place when there were more than 10 bad channels.

The Holy Grail for the quantitative EEG based psychiatry is finding the biomarkers of particular psychiatric disorders based on the measured electrical activity of the brain. We proposed some idea to find such biomarkers in Wojcik et al. (2018b) by using the frequency band analysis and estimating the most active BAs in an above-mentioned way. Some research was also reported in Zolubak et al. (2019) where authors were investigating low frequency markers in neurofeedback therapy. But indicating the most active BAs can be not enough. In the last decades, we can, however, observe the rapid growth of data science methods applied to big datasets. One of the most important of them are machine learning tools and our idea was to check whether applying different classifiers to our results, both in broadband and specific frequency band analysis, could shed some light on solving diagnoses problems. If there is a secret code of particular disorders to be found in EEG activity—the application of machine learning tools, like classifiers, seems to be the best way to decode this. As the input to classifiers, the activity of BAs in the spectrum of the mean electric charge flowing through them should be considered. Because our patients' group consisted of only 30 subjects and with a wide range of disorders it was impossible to design classifiers that could distinguish the particular disorder from the another. However, the possibility of distinguishing the reward cortical states from the punishment activity was investigated and the efficiency of selected classifiers will be discussed in the following sections to some extent.

## Results

For each participant from both the patient and control groups, we estimated the most active BAs in each EEG frequency band during the reward and punishment phases of the IGT experiment.

Thus the most active BAs for the reward variant in the patient group are presented in Table 1 and for the punishment in Table 2. The mean electric charge ι flowing through particular BA when receiving a reward by a patient is shown in Table 3 and for punishment in Table 4.

**Table 1:** Most active BA in particular subjects of the patients' group while receiving a reward during the IGT experiment in the full and in the alpha, beta, gamma, delta, and theta EEG bands.

**No**.	**Diag**.	**Full band**	**α**	**β**	**γ**	**δ**	**θ**
1	F20	BA05	BA29	BA29	BA05	BA34	BA29
2	F31	BA09	Amygd.	Amygd.	BA09	Amygd.	BA09
3	F31	BA05	Hipp.	Amygd.	BA17	Amygd.	Amygd.
4	F32.1	BA36	BA17	Amygd.	BA17	Amygd.	Amygd.
5	F32.1	BA17	BA05	Amygd.	BA17	Amygd.	BA05
6	F32.1	BA05	Amygd.	BA36	BA05	BA36	BA17
7	F32.1	BA17	Amygd.	Amygd.	BA17	BA36	Amygd.
8	F32.1	BA17	Amygd.	BA09	BA09	BA34	BA09
9	F40	Amygd.	Amygd.	Amygd.	Amygd.	BA05	BA04
10	F40	BA09	BA29	BA29	BA09	BA08	BA34
11	F40	BA46	BA24	BA36	Amygd.	BA05	BA46
12	F41	BA09	BA09	BA05	BA09	Amygd.	BA09
13	F41	Amygd.	Amygd.	BA29	Amygd.	BA29	Amygd.
14	F41	Amygd.	Amygd.	Amygd.	Amygd.	Amygd.	BA17
15	F41	BA09	Amygd.	Amygd.	BA09	Amygd.	BA04
16	F41	BA05	BA05	BA05	BA05	Amygd.	BA09
17	F41	Amygd.	Amygd.	Amygd.	Amygd.	Amygd.	BA17
18	F41	Amygd.	BA45	BA45	BA17	BA45	BA45
19	F41	BA09	Amygd.	Amygd.	BA04	Amygd.	BA09
20	F41	BA27	Amygd.	Amygd.	BA34	Amygd.	Amygd.
21	F41	Amygd.	Amygd.	Amygd.	Amygd.	Amygd.	BA04
22	F41	BA04	Amygd.	Amygd.	BA09	Amygd.	BA09
23	F41	BA05	BA36	BA27	BA05	BA27	BA05
24	F51.1	Amygd.	Amygd.	Amygd.	BA05	BA05	Amygd.
25	F51.1	BA17	BA05	BA36	BA17	BA36	BA17
26	F84.5	Amygd.	Amygd.	Amygd.	BA04	Amygd.	Amygd.
27	F84.5	BA09	BA17	BA17	BA09	BA17	BA09
28	F84.5	BA45	Hipp.	Hipp.	BA45	BA45	BA45
29	F84.5, F42	BA04	BA05	BA05	Amygd.	Amygd.	Amygd.
30	F84.5, F42	BA45	BA45	BA05	BA09	BA05	BA45

*“Amygd.” indicates Amygdala, “Hipp.” for Hippocampus areas. For detail see Discussion section in text*.

**Table 2:** Most active BA in particular subjects of the patients' group while receiving a punishment during the IGT experiment in the full and in the alpha, beta, gamma, delta, and theta EEG bands.

**No**.	**Diag**.	**Full band**	**α**	**β**	**γ**	**δ**	**θ**
1	F20	BA05	BA34	BA29	BA05	BA34	BA29
2	F31	BA09	Amygd.	Amygd.	BA09	Amygd.	Amygd.
3	F31	Amygd.	Amygd.	Amygd.	BA17	Amygd.	Amygd.
4	F32.1	BA17	BA05	Amygd.	Amygd.	Amygd.	BA17
5	F32.1	BA05	Amygd.	BA05	BA17	BA05	Amygd.
6	F32.1	BA36	Amygd.	Amygd.	BA17	BA36	BA17
7	F32.1	Amygd.	Amygd.	Amygd.	Amygd.	Amygd.	Amygd.
8	F32.1	BA17	BA09	BA09	BA09	BA34	BA09
9	F40	Amygd.	BA05	BA04	Amygd.	BA05	BA17
10	F40	BA09	BA29	BA29	BA09	BA29	BA17
11	F40	BA05	Amygd.	BA36	Amygd.	Amygd.	BA17
12	F41	Amygd.	Amygd.	BA05	BA09	Amygd.	BA05
13	F41	BA04	Amygd.	BA29	BA05	BA29	Amygd.
14	F41	BA43	BA17	BA05	Amygd.	BA36	BA17
15	F41	Amygd.	BA05	Amygd.	Amygd.	Amygd.	Amygd.
16	F41	Amygd.	BA05	BA05	BA05	Amygd.	BA09
17	F41	Amygd.	Amygd.	Amygd.	Amygd.	Amygd.	Amygd.
18	F41	Amygd.	BA45	BA45	Amygd.	BA45	BA45
19	F41	Amygd.	Amygd.	Amygd.	Amygd.	Amygd.	Amygd.
20	F41	BA27	Amygd.	Amygd.	BA23	Amygd.	BA17
21	F41	BA04	Amygd.	Amygd.	BA09	Amygd.	BA04
22	F41	BA04	Amygd.	Amygd.	Amygd.	Amygd.	Amygd.
23	F41	BA05	BA05	BA27	Amygd.	BA41	BA05
24	F51.1	BA05	Amygd.	Amygd.	Amygd.	Amygd.	Amygd.
25	F51.1	BA05	BA05	BA34	BA17	Amygd.	BA17
26	F84.5	Amygd.	Amygd.	Amygd.	BA05	Amygd.	BA05
27	F84.5	BA09	Amygd.	BA17	BA09	BA17	BA09
28	F84.5	BA41	Hipp.	Hipp.	BA45	BA31	BA45
29	F84.5, F42	Amygd.	BA05	Amygd.	Amygd.	Amygd.	BA05
30	F84.5, F42	BA05	BA05	BA05	BA05	BA05	BA05

*“Amygd.” indicates Amygdala, “Hipp.” for Hippocampus areas. For detail see Discussion section in text*.

**Table 3:** The ι for the most active BA in particular patients receiving a reward during the IGT experiment obtained from the sLORETA quantitative analysis.

**No**.	**Diag**.	**Full band**	**α**	**β**	**γ**	**δ**	**θ**
		**ι [μC]**	**ι [μC]**	**ι [μC]**	**ι [μC]**	**ι [μC]**	**ι [μC]**
1	F20	25.45	8.60	7.49	30.96	9.47	7.12
2	F31	45.10	3.99	5.59	43.32	6.03	17.82
3	F31	11.77	2.82	3.54	14.82	5.55	5.60
4	F32.1	37.92	3.40	33.19	38.90	11.31	6.01
5	F32.1	77.11	20.28	7.56	38.91	9.94	15.80
6	F32.1	200.70	15.17	19.93	56.61	17.39	20.45
7	F32.1	55.50	11.87	12.61	30.02	13.26	6.03
8	F32.1	24.65	4.58	9.10	69.61	8.19	8.05
9	F40	19.24	18.92	14.47	23.05	9.04	6.61
10	F40	46.20	5.35	5.60	20.27	2.67	7.69
11	F40	20.49	2.88	3.81	16.15	3.55	6.82
12	F41	67.16	6.03	10.13	20.88	14.89	6.40
13	F41	48.25	6.29	25.76	45.79	22.14	6.50
14	F41	118.70	5.40	6.24	50.14	9.89	20.32
15	F41	55.71	4.41	3.95	32.42	4.74	6.36
16	F41	68.59	11.27	11.07	44.56	8.43	16.58
17	F41	81.21	14.93	20.20	53.17	19.26	14.13
18	F41	45.66	8.50	15.83	39.21	14.15	15.55
19	F41	40.30	3.68	3.75	44.72	4.55	11.12
20	F41	91.03	9.34	11.66	93.58	16.13	7.46
21	F41	67.74	5.46	8.14	45.63	8.99	10.91
22	F41	26.20	6.75	10.53	61.26	14.79	9.62
23	F41	29.25	4.96	8.02	20.15	11.23	6.23
24	F51.1	34.89	2.46	3.56	17.63	3.06	5.62
25	F51.1	28.82	2.14	3.22	20.67	3.49	4.06
26	F84.5	27.53	4.15	5.82	8.55	7.11	6.88
27	F84.5	44.27	2.05	6.32	28.75	7.86	5.04
28	F84.5	20.34	6.18	5.89	37.28	2.03	14.97
29	F84.5, F42	15.99	5.19	6.85	16.47	4.32	5.52
30	F84.5, F42	27.07	5.95	33.39	34.75	26.89	10.16

*For detail see the Discussion section in the text*.

**Table 4:** The ι for the most active BA in particular patients receiving a punishment during the IGT experiment obtained from the sLORETA quantitative analysis.

**No**.	**Diag**.	**Full band**	**α**	**β**	**γ**	**δ**	**θ**
		**ι [μC]**	**ι [μC]**	**ι [μC]**	**ι [μC]**	**ι [μC]**	**ι [μC]**
1	F20	58.78	8.41	11.80	19.74	14.19	13.74
2	F31	36.15	5.56	9.09	62.91	9.05	11.46
3	F31	19.13	3.33	10.09	16.76	10.48	5.27
4	F32.1	70.61	9.02	29.37	35.87	28.28	19.50
5	F32.1	44.21	39.90	13.93	44.60	20.02	25.72
6	F32.1	86.34	29.29	40.46	62.67	31.10	33.35
7	F32.1	70.96	14.87	17.38	39.71	19.95	25.57
8	F32.1	88.81	13.66	15.96	54.23	21.21	22.31
9	F40	33.17	26.94	25.38	22.23	20.06	6.64
10	F40	117.98	9.74	8.07	68.39	6.65	11.47
11	F40	30.50	5.26	7.00	22.34	8.58	8.03
12	F41	99.30	9.41	17.07	49.72	28.45	9.55
13	F41	49.64	6.88	43.05	37.35	46.80	12.43
14	F41	231.56	11.06	11.79	87.43	18.20	15.64
15	F41	103.02	8.47	8.90	91.82	12.95	15.18
16	F41	100.32	15.01	19.75	54.24	15.90	22.40
17	F41	84.65	20.36	26.41	64.05	37.07	47.20
18	F41	148.46	19.03	32.64	74.02	28.62	28.91
19	F41	57.55	6.54	5.95	39.59	9.03	18.25
20	F41	134.75	13.39	16.48	77.11	24.33	16.96
21	F41	117.67	9.80	12.43	87.12	18.07	10.34
22	F41	69.91	12.87	17.81	83.47	25.76	23.91
23	F41	77.93	10.15	12.17	48.43	18.50	15.37
24	F51.1	28.63	6.77	5.90	25.05	5.29	10.72
25	F51.1	22.51	4.22	6.80	25.58	8.54	7.28
26	F84.5	21.58	6.90	8.32	9.72	9.31	5.00
27	F84.5	76.76	5.10	12.97	92.33	16.31	21.26
28	F84.5	35.00	9.39	8.56	49.69	3.91	22.36
29	F84.5, F42	56.93	6.52	11.55	1.37	8.46	9.62
30	F84.5, F42	121.72	11.22	55.63	39.35	51.99	12.92

*For detail see the Discussion section in the text*.

In analogy for the control group the reward associated most active BAs are presented in Table 5 and the punishment responses in Table 6. Tables 7, 8 present the values of ι calculated for each member of the control group in the reward and punishment variants of response, respectively.

**Table 5:** The most active BA in particular subjects of the control group while receiving a reward during the IGT experiment in the full and in the alpha, beta, gamma, delta, and theta EEG bands.

**No**.	**Full band**	**α**	**β**	**γ**	**δ**	**θ**
1	Amygd.	BA05	BA05	Amygd.	BA05	BA05
2	Amygd.	Amygd.	Amygd.	Amygd.	Amygd.	Amygd.
3	BA17	BA05	BA17	BA09	Hipp.	BA09
4	BA17	Amygd.	BA36	BA17	BA36	BA17
5	BA09	BA05	BA05	BA09	BA27	BA09
6	BA17	Amygd.	Amygd.	BA17	Amygd.	BA17
7	BA17	Amygd.	Amygd.	BA09	Amygd.	BA09
8	BA05	Amygd.	BA05	BA05	BA05	BA09
9	BA09	BA18	BA24	BA17	BA18	BA17
10	BA28	BA34	BA34	BA09	BA34	Amygd.
11	BA05	Amygd.	BA05	BA05	BA05	BA05
12	BA09	BA46	BA05	BA09	Amygd.	BA09
13	BA17	BA17	BA17	BA17	BA17	BA17
14	BA09	Amygd.	Amygd.	BA09	Amygd.	BA17
15	BA09	Amygd.	BA27	BA09	BA27	BA17
16	BA09	BA05	Amygd.	BA09	Amygd.	BA05
17	BA09	BA29	BA29	BA17	BA28	BA17
18	BA05	Amygd.	BA34	BA09	Amygd.	BA09
19	Amygd.	BA04	BA04	Amygd.	BA04	Amygd.
20	BA09	Amygd.	Amygd.	BA09	Amygd.	Amygd.
21	BA17	Amygd.	BA36	BA17	BA36	BA17
22	Amygd.	Amygd.	Amygd.	Amygd.	BA28	Amygd.
23	Hipp.	BA17	BA27	BA17	BA27	BA17
24	BA41	Amygd.	Amygd.	BA17	BA05	Amygd.
25	BA17	BA42	BA42	BA17	BA27	BA17
26	BA09	BA09	Amygd.	BA09	Amygd.	BA17
27	BA05	BA09	Amygd.	Amygd.	Amygd.	BA09
28	BA46	Amygd.	Amygd.	Amygd.	BA05	BA46
29	Amygd.	BA45	BA27	BA17	BA27	BA45
30	BA17	BA05	BA05	BA17	BA36	Amygd.

*“Amygd.” indicates Amygdala, “Hipp.” the Hippocampus areas*.

**Table 6:** The most active BA in particular subjects of the control group while receiving a punishment during the IGT experiment in the full and alpha, beta, gamma, delta, and theta EEG bands.

**No**.	**Full band**	**α**	**β**	**γ**	**δ**	**θ**
1	Amygd.	BA05	BA05	Amygd.	BA05	BA05
2	BA05	Amygd.	Amygd.	Amygd.	Amygd.	Amygd.
3	BA45	BA09	BA41	BA17	BA17	BA09
4	BA05	BA45	Amygd.	BA17	BA36	BA17
5	BA05	BA05	BA05	Amygd.	BA27	BA09
6	BA09	BA17	Amygd.	BA09	Amygd.	BA17
7	BA09	Amygd.	Amygd.	Amygd.	Amygd.	BA09
8	Amygd.	Amygd.	Amygd.	Amygd.	Amygd.	Amygd.
9	BA17	BA07	BA18	BA17	BA18	BA17
10	BA29	BA17	BA34	Amygd.	BA34	BA17
11	BA05	Amygd.	BA05	BA05	BA05	Amygd.
12	BA04	Amygd.	BA05	BA09	Amygd.	BA09
13	BA17	BA17	BA17	BA17	BA41	BA17
14	BA05	Amygd.	BA27	BA05	BA41	Amygd.
15	BA09	Amygd.	Amygd.	BA17	BA28	BA09
16	Amygd.	BA05	Amygd.	BA09	Amygd.	BA05
17	BA09	BA29	BA29	BA09	BA28	BA05
18	BA09	BA04	Amygd.	BA09	BA34	BA09
19	BA05	BA04	BA29	Amygd.	BA04	Amygd.
20	Amygd.	Amygd.	Amygd.	BA17	Amygd.	Amygd.
21	BA17	BA17	BA36	BA17	BA36	BA17
22	BA17	Amygd.	BA28	BA17	BA28	BA17
23	BA17	BA17	BA05	BA17	BA27	BA17
24	BA41	Amygd.	Amygd.	BA17	Amygd.	Amygd.
25	BA17	BA42	BA42	BA17	BA27	BA05
26	BA09	Amygd.	BA09	BA09	Amygd.	Amygd.
27	BA05	Amygd.	BA05	Amygd.	BA05	Amygd.
28	BA05	Amygd.	Amygd.	BA05	Amygd.	BA05
29	BA09	BA05	BA27	BA09	Amygd.	BA17
30	Amygd.	BA05	BA05	BA17	BA36	Amygd.

*“Amygd.” indicates Amygdala, “Hipp.” the Hippocampus areas*.

**Table 7:** The ι for the most active BA in particular subjects of the control group receiving a reward during the IGT experiment obtained from the sLORETA quantitative analysis.

**No**.	**Full band**	**α**	**β**	**γ**	**δ**	**θ**
	**ι [μC]**	**ι [μC]**	**ι [μC]**	**ι [μC]**	**ι [μC]**	**ι [μC]**
1	35.13	4.97	6.25	21.43	8.76	5.09
2	123.05	20.57	11.64	157.64	5.93	42.89
3	18.50	2.29	3.12	30.77	3.75	6.08
4	28.76	3.10	2.37	49.85	3.44	6.78
5	34.17	4.95	6.11	45.50	3.89	13.49
6	35.07	2.95	4.85	56.67	3.46	11.16
7	44.39	4.44	4.68	41.99	4.94	9.71
8	44.52	2.57	2.82	27.37	3.55	5.54
9	22.01	2.59	3.50	44.04	5.46	4.52
10	29.10	3.58	5.32	36.85	6.92	4.49
11	74.89	2.90	8.60	43.62	7.82	3.83
12	16.20	2.82	2.76	42.64	2.88	6.58
13	91.48	4.22	4.35	93.37	1.06	7.83
14	26.73	6.71	7.43	17.22	1.06	6.80
15	31.23	3.03	5.06	69.70	6.23	11.95
16	40.49	2.96	2.81	25.40	2.68	5.86
17	19.58	5.02	9.06	9.08	4.37	5.10
18	29.44	5.69	7.51	20.83	3.24	6.22
19	20.94	12.05	10.69	27.52	4.98	17.75
20	36.71	2.56	4.77	26.71	2.23	5.51
21	36.85	4.48	3.62	40.97	6.17	9.02
22	22.35	2.60	4.17	11.91	4.64	4.07
23	25.54	9.62	6.41	60.50	6.38	16.57
24	93.76	4.25	5.60	13.82	5.45	6.00
25	28.75	9.77	18.81	27.34	6.59	6.99
26	41.20	6.59	8.00	89.68	8.19	14.10
27	77.02	5.40	10.27	95.49	6.93	13.58
28	16.18	2.64	3.44	18.24	3.72	4.03
29	19.45	2.88	2.76	26.82	2.98	4.51
30	22.57	16.39	20.27	30.82	7.50	7.19

*For detail see the Discussion section in the text*.

**Table 8:** The ι for the most active BA in particular subjects of the control group receiving a punishment during the IGT experiment obtained from the sLORETA quantitative analysis.

**No**.	**Full band**	**α**	**β**	**γ**	**δ**	**θ**
	**ι [μC]**	**ι [μC]**	**ι [μC]**	**ι [μC]**	**ι [μC]**	**ι [μC]**
1	47.89	8.94	11.78	63.56	15.64	8.99
2	203.19	27.55	22.29	277.38	10.28	75.45
3	34.32	4.23	19.78	21.88	5.85	5.68
4	34.02	3.95	4.14	24.11	4.80	12.66
5	41.10	8.24	12.84	54.96	6.38	15.80
6	80.51	7.69	8.61	47.67	9.31	18.62
7	55.34	7.10	8.21	29.96	9.99	15.50
8	125.22	4.56	5.83	63.70	7.58	8.83
9	29.85	5.90	6.48	37.00	9.27	11.87
10	89.41	8.66	10.62	82.42	12.51	7.70
11	132.74	6.12	11.68	109.95	13.24	6.27
12	34.78	5.58	4.91	40.29	4.90	6.63
13	203.37	6.69	6.21	89.38	0.99	31.37
14	37.34	14.02	11.15	34.81	1.80	14.62
15	51.69	10.40	13.26	35.51	17.34	11.14
16	31.28	4.35	4.32	17.42	6.62	8.93
17	46.04	6.65	13.57	14.03	6.91	5.08
18	50.01	7.94	11.22	78.38	6.83	12.96
19	39.11	11.51	14.88	40.65	8.03	16.86
20	45.93	2.76	3.95	34.68	4.67	6.29
21	68.19	10.09	7.55	73.64	10.18	15.74
22	34.54	4.33	7.62	29.19	10.21	6.92
23	58.34	18.68	12.75	75.45	9.00	18.49
24	118.22	9.63	11.49	64.50	11.44	14.56
25	70.20	17.48	19.10	55.03	10.83	9.88
26	54.22	9.57	13.28	103.73	13.20	23.43
27	158.88	8.50	11.97	71.10	10.81	16.73
28	26.53	4.51	5.84	15.03	5.85	5.23
29	32.70	3.66	4.68	34.81	5.66	7.71
30	45.49	24.36	30.51	34.31	13.03	14.36

*For detail see the Discussion section in the text*.

As follows from Tables 1–8 in all cases the Amygdala is hyperactive and the order of the value of ι tends to be similar for reward and punishment in both the subject and control groups in each frequency band.

**Figure 4:**
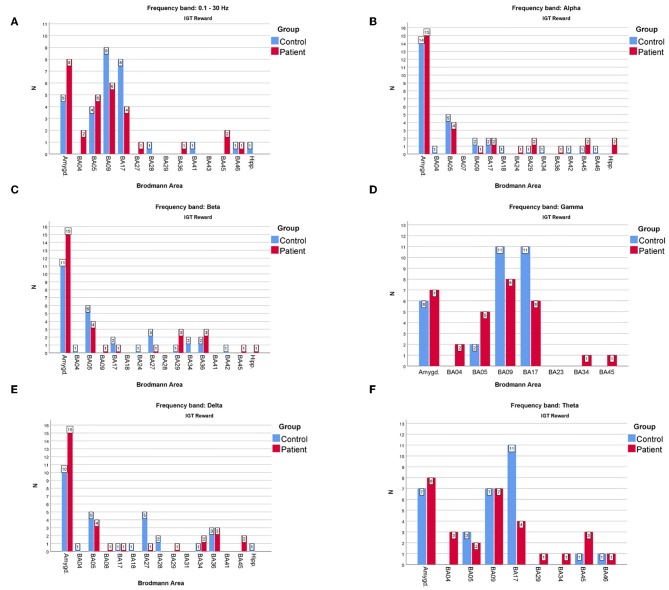
The comparison of the most frequently active BAs in the patients and the control groups in the “reward” variant of IGT response for **(A)**—full, **(B)**—alpha, **(C)**—beta, **(D)**—gamma, **(E)**—delta, **(F)**—theta frequency bands. The y-axis refers to the N—number of subjects with particular BA maximum activity noted.

**Figure 5:**
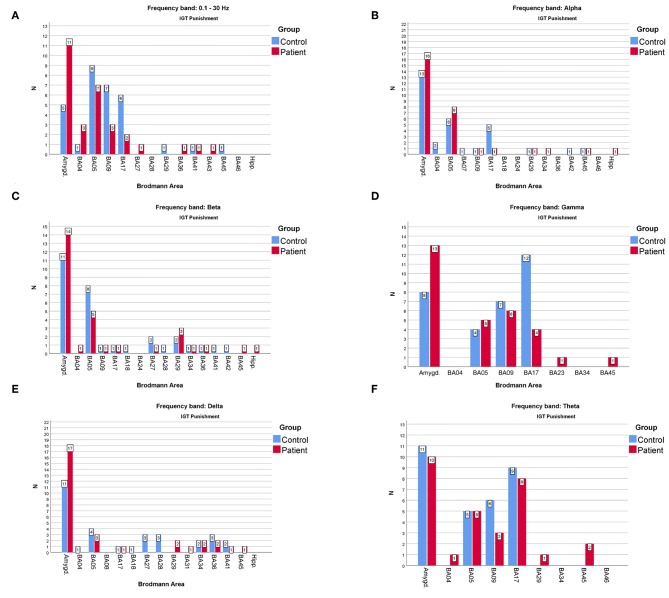
The comparison of the most frequently active BAs in the patients and the control groups in the “punishment” variant of IGT response for **(A)**—full, **(B)**—alpha, **(C)**—beta, **(D)**—gamma, **(E)**—delta, **(F)**—theta frequency bands. The y-axis refers to the N—number of subjects with particular BA maximum activity noted.

However, the comparison shown in Figure 4 for the rewards and in Figure 5 for the punishment can shed some light on the main differences in cortical responses of people with psychiatric disorders and members of the control group.

As far as the frequency of BA appearances in the IGT reward cortical responses are concerned (Figure 4) one can note that:

- The one of the amygdala is significantly higher in the patients compared to the control group when observed in the full, beta and delta frequency bands.
- The one of BA17 is significantly higher in the control group than in the patients when observed in the gamma and theta bands.
- The one of BA09 is higher in the control compared to the patients' group in the full and gamma frequency bands.

When considering the frequency of BA appearance in the IGT punishment responses one can observe that:

- The one of the amygdala is significantly higher in the patients than in the control group members in the full, alpha, beta, delta and gamma frequency bands.
- The one of BA17 is significantly higher in the control than in the patients' group in the full and gamma bands.
- The BA09 is significantly higher in the control group members than in patients when looking at the full and theta frequency bands.
- The BA05 in the control group is higher than in the patients in the full and beta frequency bands while in the alpha band it is lower.

The role of the amygdala during the decision-making process was discussed before even by the authors of IGT (Bechara et al., 2003). It is known that both the amygdala and orbitofrontal cortex are parts of a neural circuit critical for judgement and decision-making being under influence of “primary inducers” defined as stimuli that unconditionally, or through learning (e.g., conditioning and semantic knowledge), can (perceptually or subliminally) produce states that are pleasurable or aversive (Bechara et al., 2003).

In order to verify the somatic marker hypothesis which proposes that decision-making is a process depending on emotion, some research of the destroyed amygdala was carried out (Bechara et al., 1999; Gupta et al., 2011). During some fMRI studies it was shown that amygdala influences the decision-making process in the risk-taking experiments involving information ambiguity (Hsu et al., 2005).

Our experiments show that the people with psychiatric disorders have the amygdala more frequently hyperactive when compared to healthy participants from the control group.

The Azure Machine Learning Studio was used to construct seven different classifiers and next to compare their efficiency in the reward/punishment characteristic cortical activity detection and classification tasks. Our classifiers were designed in order to separate two classes (reward/punishment) and were as follow: logistic regression, decision jungle, support vector machine, boosted decision tree, averaged perceptron, Bayes point machine, classic neural network and locally-deep support vector algorithms. Each classifier had its own characteristics which are shown in Table 10. The registered activity of particular BAs manifesting itself by the mean electric charge ι in all discussed EEG frequency bands, including the broadband was taken as inputs to the classifier.

Under ideal conditions, it would be expected to construct effective classifiers for particular psychiatric disorders, but having only 30 diagnosed subjects in our cohort with so many different diagnoses is far from being enough to perform any statistics. For the machine learning tasks, the control group was extended by an additional 11 healthy males finally reaching 41 subjects. Thus, the joined cohort consisted of 41 healthy males and 30 males with some disorders, in a total of 71 participants. Note that in typical ERP experiments there are standard (STD) and target (TGT) stimuli. In the case of IGT, the punishment is treated as STD and the reward is TGT as practically everyone dares to win. For each participant registered the reward and punishment states, finally giving 2 × 71 = 142 averaged responses to the investigated set.

The 5-fold Cross-Validation method was used to validate the efficiency of all classifiers.

Then the values of classification accuracy, recall and precision were calculated and results are presented in Table 9.

**Table 9:** Comparison of the discussed classifiers efficiency for all frequency bands, including the broadband in the STD (punishment) and TGT (reward) classification tasks.

**Broad-band**	**Corr. TGT**	**Inc. TGT**	**Corr. STD**	**Inc.STD**	**Acc**.	**Recall**	**Prec**.
Logistic regression	55	32	39	16	0.662	0.549	0.709
Decision jungle	42	31	40	29	0.577	0.563	0.580
Support vector machine	35	25	46	36	0.561	0.521	0.698
Avg. perceptron classifier	53	32	39	18	0.684	0.549	0.684
Bayes point machine	33	15	56	38	0.627	0.789	0.596
Neural network	55	41	30	16	0.652	0.423	0.652
Locally-deep support vector	55	34	37	16	0.698	0.648	0.561
**α**							
Logistic regression	54	27	44	17	0.690	0.620	0.721
Decision jungle	48	27	44	23	0.648	0.620	0.657
Support vector machine	41	29	42	30	0.585	0.577	0.683
Avg. perceptron classifier	51	28	43	20	0.662	0.606	0.683
Bayes point machine	46	28	43	25	0.627	0.606	0.632
Neural network	61	37	34	10	0.669	0.479	0.773
Locally-deep support vector	41	29	42	30	0.585	0.592	0.583
**β**							
Logistic regression	51	23	48	20	0.697	0.676	0.706
Decision jungle	51	24	47	20	0.690	0.662	0.701
Support vector machine	53	26	45	18	0.690	0.634	0.714
Avg. perceptron classifier	49	24	47	22	0.676	0.662	0.681
Bayes point machine	35	13	58	36	0.655	0.817	0.617
Neural network	58	36	35	13	0.655	0.493	0.729
Locally-deep support vector	46	29	42	25	0.620	0.592	0.627
**γ**							
Logistic regression	52	33	38	19	0.634	0.535	0.667
Decision jungle	42	30	41	29	0.585	0.577	0.586
Support vector machine	52	29	42	19	0.662	0.592	0.689
Avg. perceptron classifier	49	32	39	22	0.620	0.549	0.639
Bayes point machine	34	17	54	37	0.620	0.761	0.593
Neural network	50	33	38	21	0.620	0.535	0.644
Locally-deep support vector	41	31	40	30	0.570	0.563	0.571
**δ**							
Logistic regression	56	28	43	15	0.697	0.606	0.741
Decision jungle	49	23	48	22	0.683	0.775	0.743
Support vector machine	56	27	44	15	0.704	0.620	0.746
Avg. perceptron classifier	56	27	44	15	0.704	0.620	0.746
Bayes point machine	39	14	57	32	0.676	0.803	0.640
Neural network	62	37	34	9	0.676	0.479	0.791
Locally-deep support vector	50	28	43	21	0.655	0.606	0.672
**θ**							
Logistic regression	53	24	47	18	0.704	0.662	0.723
Decision jungle	51	31	40	20	0.641	0.563	0.667
Support vector machine	50	26	45	21	0.669	0.521	0.569
Avg. perceptron classifier	45	27	44	26	0.627	0.620	0.629
Bayes point machine	29	14	57	42	0.606	0.803	0.576
Neural network	61	41	30	10	0.641	0.423	0.750
Locally-deep support vector	50	26	45	21	0.669	0.521	0.569

*Note, that numbers in rows sum to 142—for detail see text*.

**Table 10:** Characteristics of the Two-Class classifiers used in IGT analysis.

**Two-class logistic regression**	
Optimization tolerance	0.0001
L1 weight	0.1
L2 weight	0.1
Memory size	11
Quiet	True
Use threads	True
Allow unknown levels	True
**Two-class decision jungle**	
Ensemble element count	8
Max. depth	32
Max. width	128
Optimization step count	2
Resampling method	Bagging
Random number seed	5
Allow unknown levels	True
**Two-class support vector machine**	
Number of iterations	101
Lambda	1.0
Normalize features	True
Perform projection	False
Allow unknown levels	True
**Two-class average perceptron classifier**	
Batch size	256
Initial learning rate	0.1
Learning rate decay exponent	0.5
Averaging weight	0.5
Tolerance	1E-05
Maximum number of iterations	101
Allow unknown levels	True
**Two-class bayes point machine**	
Allow unknown levels	True
Random number seed	2,342
Training iteration count	30
Add bias	True
**Two-class neural network**	
Loss function	CrossEntropy
Is initialized from string	False
Is classification	False
Initial weights diameter	0.1
Learning rate	0.1
Momentum	0
Data normalizer type	MinMax
Number of input features	88
Number of hidden nodes	100
Number of iterations	51
Shuffle	True
Allow unknown levels	True
**Two-class locally-deep support vector**	
Tree depth	2
Lambda W	0.1
Lambda theta	0.1
Lambda theta prime	0.1
Sigma	1
Number of iterations	14,500
Normalizer type	MinMax
Allow unknown levels	True

As one can see in Table 9 there is no ideal classifier that could be applied to all of the EEG frequency bands, including the broadband.

For the broadband, the best results were achieved by the Locally-Deep Support Vector (acc. 0.698) and the Average Perceptron Classifier (acc. 0.684) methods.

In the α band the Logistic Regression (acc. 0.690) and Neural Network (acc. 0.669) turned out to be the best classifiers

When one looks at classifiers' results in the β band he notes the Logistic Regression (acc. 0.697), Decision Jungle (acc. 0.690) and Support Vector Machine (0.690) as the best, however the Logistic Regression has the highest recall value of 0.676, while the highest precision of 0.714 is achieved by Support Vector Machine.

If one studies the activity in the γ band he finds the highest efficiency for the Support Vector Machine (acc. 0.662) and again Logistic Regression (acc. 0.634).

For the δ band the highest accuracy 0.704 was achieved by the Support Vector Machine and Average Perceptron Classifier.

In case of the θ band, the best three ones were Logistic Regression (acc. 0.704), Support Vector Machine (0.669), and Locally Deep Support Vector (0.669), the Logistic Regression with the highest precision 0.723.

Note that the Bayes Point Machine did not perform well in any of EEG frequency bands.

## Discussion

In our experiment the relations between the decision-making process and the emotional responses given by the soma under such experimental conditions are also visible. Somatosensory association cortex (SAC) is mentioned in some papers on decisions making where it is even stated that somatosensory pattern marks the scenario as good or bad (Bechara et al., 2000; Donner et al., 2009). Our results also show that BA05 is one of the few most frequently active BAs in the patients and the control groups members., Moreover, the activity is qualitatively different in different frequency bands.

As well the dorsolateral prefrontal cortex (BA09) is often reported as engaged in decision-making processes. It was even found that damage of this structure results in poor performance in IGT (Fellows and Farah, 2004) and the fMRI studies have shown that the dorsolateral prefrontal cortex plays a role of negotiator establishing the link among sensory evidence, decision, and action during the decision making (Heekeren et al., 2006). Hyperactive BA09 is also reported to be found in other cognitive processes (Elliott, 2003), like working memory (Barbey et al., 2013), cognitive flexibility (Monsell, 2003), and planning (Chan et al., 2008). In our experiments the BA09 seems to be much more active in the control group when compared to the patients.

The visual processing areas provide the sensory evidence for a decision (Heekeren et al., 2004) and our results confirmed that the primary visual cortex is one of the most engaged areas in such processes, much more active in the control than in the patients' group. Some experiments involve the visual motion detection in the decision-making process among macaques (Huk and Shadlen, 2005) and this can be an inspiration for our future research.

The research protocol has been proposed to record the electroencephalographical cortical activity of the human brain during the decision making process. We chose the IGT as one of the tasks that are most often used to investigate people making decisions. The sLORETA was then applied to find the most frequently active BA in the brain cortex both in the patients and the control group.

Some attempts to find biomarkers in the quantitative EEG signals were made for example by John et al. (1988). The frequency band analysis is often used in real-time computing of the engagement index (Lubar et al., 1995; Pope et al., 1995; Chaouachi et al., 2010). Moreover, some cognitive functions in patients with psychiatric disorders are different from those in healthy members of control groups (Trivedi, 2006).

Even though the cohorts were not large we could prove some findings reported after performing such experiments by means of much more sophisticated techniques including fMRI. We had 30 subjects with several different diagnoses. That is why it is hard to apply any more sophisticated statistical analysis. Collecting neurophysiological data is a real challenge for neuroinformatics (Bigdely-Shamlo et al., 2016; Cavanagh et al., 2017). In future it would require building separate cohorts for each particular disorder, for all genders and age ranges. Then it would be possible to make quantitative comparisons of cortical activity which hopefully could even lead to building psychiatric disorders classifiers.

The additional aim of this paper was to check whether it is possible to assess without looking into logs the subject's response in the IGT experiments using only the brain cortical electric activity as the input to the algorithm. The effectiveness of seven different tools from the Azure Machine Learning environment was investigated. The summary of the results is presented in Table 9.

It was shown that there is no universal classifier for each frequency band. However, for the future analysis the Logistic Regression in the α, β, and θ bands should be considered as well as the Support Vector Machine in the β, γ, and δ. Very interesting behavior can be observed for the Averaged Perceptron Classifier in the δ band which together with the Support Vector Machine has one the best recall and precision characteristics in the discussed research.

It is expected that for the larger dataset the efficiency would be much higher. This is the initial stage of our research but one can take it for granted that tuning-up the parameters would also improve the method performance. Now it is hard to predict which methods would be best for additional improving such classifiers. Probably the applications of sophisticated tools offered by applied mathematics (Kakiashvili et al., 2012; Koczkodaj and Szybowski, 2015) or even solutions found for engineering applications in computer science (Bolanowski and Paszkiewicz, 2015; Grabowski et al., 2015) along with big data analysis in such case could add some value.

At this stage, we had access to a limited number of patients. In our methodology, we decided to choose only those who had not taken any psychotropic medicines before. The aim of the research presented in this paper was to show the way in which the biomarkers can be searched. Putting the representatives of several disorders into one group by some readers can be recognized as controversial. On the other hand, we did not intend to give the final answer and to satisfy the definition of a biomarker in the full range of its properties. This would require at least 30 cases for each disorder and if one takes into consideration males and females, different age ranges, handedness—we get the number of about 400 patients for one problem, not saying about the control group. Consideration only the one disorder based on several patients does not make much sense as it would be hard to do the serious statistical analysis. But our results show that there can be quantitative methods to start the hunt for psychiatric disorders biomarkers.

Remembering that the interview is still the most important tool used in current psychiatry we are aware of the fact that developing tools and methods able to support the psychiatrist in the process of diagnosing are in a great demand and would improve the comfort of patients' life in the future.

## Data Availability Statement

The raw data supporting the conclusions of this manuscript will be made available by the authors, without undue reservation, to any qualified researcher.

## Ethics Statement

This study was carried out in accordance with the recommendations of Guidelines for Good Clinical Practice (GCP). The protocol was approved by the Medical University of Lublin Bioethical Commission. All subjects gave written informed consent in accordance with the GCP. Permission No. KE-0254/138/2015 and No. KE-0254/140/2015 given by the Medical University of Lublin Bioethical Commission on May 28th, 2015.

## Author Contributions

GW: project idea and coordination, experiment design, subjects' recruitment, interpretation of results. JM: project idea, experiment design, subjects' recruitment, psychiatric diagnosis, interpretation of results. AK: work in laboratory, cleaning signal, computations, statistical analysis. PS, FP, and LK: statistical analysis, writing scripts, work in laboratory, cleaning signal. AG-B: work in laboratory.

### Conflict of Interest

The authors declare that the research was conducted in the absence of any commercial or financial relationships that could be construed as a potential conflict of interest.

## Footnotes

^1^GeoSource 3 electrical source imaging packages: https://www.egi.com/research-division/electrical-source-imaging/geosource

^2^BESA: ERP analysis and averaging, http://www.besa.de/products/besa-research/features/erp-analysis-and-averaging/

^3^BESA: Source analysis and imaging, http://www.besa.de/products/besa-research/features/source-analysis-and-imaging/

^4^Electrical Geodesic Systems, Inc., 500 East 4th Ave. Suite 200, Eugene, OR, 97401, USA.

^5^Psychology Software Tools, Inc. PST, Sharpsburg Business Park, 311 23rd Street Ext., Suite 200, Sharpsburg, PA, 15215-2821, USA.
